# Causal association of immune effector proteins with sepsis: A Mendelian randomization study

**DOI:** 10.1097/MD.0000000000039494

**Published:** 2024-09-06

**Authors:** Yongsheng Wang, Chuchu Xu, Yao Zhang, Lijuan Zhou, Ting Zhang, Xiaona Yin, Xi Wang, Yinling Jiang, Fangbing Du, Xiaoqiong Wang

**Affiliations:** a Department of Respiratory and Critical Care Medicine, The Second People’s Hospital of Hefei, Hefei Hospital Affiliated to Anhui Medical University, Hefei, Anhui, China; b Fifth Clinical Medical College of Anhui Medical University, Hefei, Anhui, China; c Hefei Second People’s Hospital Affiliated With Bengbu Medical College, Hefei, Anhui, China; d Department of Cardiovascular Medicine, The Second People’s Hospital of Hefei, Hefei Hospital Affiliated to Anhui Medical University, Hefei, Anhui, China.

**Keywords:** causal relationship, GWAS, immune effector, Mendelian randomization, sepsis

## Abstract

Sepsis is an infection-induced systemic inflammatory response syndrome. Immune regulation plays a crucial role in sepsis. We looked into the link between immune effector–related proteins and sepsis in this study by using both univariate and multivariate Mendelian randomization (MR) analyses. We accessed and collected data from the Integrative Epidemiology Unit’s Open About Sepsis genome-wide association study database. The 6 immune effector–associated proteins each contained 10,534,735 single-nucleotide polymorphisms from 3301 samples. Using the weighted median, MR-Egger, simplex, inverse-variance weighting, and weighted mode methods, univariate MR then investigated the link between complement factor H-related protein-5 (CFHR5), Fc epsilon receptor II (FCER2), granzyme B (GZMB), major histocompatibility complex, class II, DQ alpha (HLA-DQA2), mannose-binding lectin 2 (MBL2), or myeloperoxidase (MPO) and sepsis. In the inverse-variance weighted results, the *P* values of all 6 immune effector–related proteins were <0.05, suggesting a possible causal relationship between them and sepsis. MBL2 (odds ratio [OR] = 1.046) was a risk factor for sepsis, while the other proteins (FCER2: OR = 0.922; GZMB: OR = 0.908; CFHR5: OR = 0.858; HLA-DQA2: OR = 0.896; MPO: OR = 0.875) were safety factors. By revealing a causal link between sepsis and CFHR5, FCER2, GZMB, HLA-DQA2, MBL2, or MPO, our study offers an essential resource for additional investigations on the subject.

## 1. Introduction

Sepsis is a serious public health event that results in significant mortality and long-term disability. Alarmingly, the risk of death for patients with sepsis increases by 7% to 8% for every hour that treatment is delayed.^[1]^ Therefore, rapid and accurate diagnosis and treatment are not only critical but also a matter of life and death. For the diagnosis of sepsis, we still use the 2016 version of Sepsis 3.0, which provides a composite score of organ function across the body, along with microbiological and biochemical tests.^[2]^ This guideline requires scoring multiple organs throughout the body, which is too slow and inaccurate for the initial screening in an emergency setting. Therefore, it is crucial to identify high-risk factors that can immediately pinpoint sepsis.

Sepsis occurs when the body’s immune response to infection becomes dysfunctional.^[3]^ Our immune system faces a conundrum: while it strives to combat infections, it becomes entangled in its own malfunction, leading to harm to its own tissues and organs. Literature reports that CD8^+^ T cells, as part of the adaptive immune system, have a more specific and sustained response. In septic patients, granzyme B (GZMB), a CD8^+^ T-cell immune effector process protein, has an elevated expression.^[4]^ mannose-binding lectin 2 (MBL2), a lectin, binds to carbohydrates on the surfaces of bacteria, viruses, and fungi. It facilitates phagocytosis by activating the complement system and generating an innate immunological response in the host.^[5]^ Patients with sepsis are closely associated with increased MBL2 expression.^[6]^ It is, therefore, crucial to explore the link between immune effector proteins and sepsis.

Mendelian randomization (MR) is a statistical method for assessing the causal effect of exposure factors on disease, using genetic variation as an instrumental variable (IV).Because it is less affected by confounding factors, it is now widely used in causality studies of disease.^[7]^ Through a review of the literature, we found that the lipoprotein-associated phospholipids,^[8]^ mitochondrial-associated proteins,^[9]^ circulating cytokines,^[10]^ and gut microbiota^[11]^ were found to be closely related to sepsis using the results of an MR study. This provides new ideas for the treatment and prevention of sepsis. However, there is a lack of sufficient genetic evidence for the effect of immune regulation–related proteins on sepsis. Therefore, there is an urgent need to investigate the close relationship using MR methods, with a view to searching for high-risk factors for sepsis from an immune perspective and to establish a simple sepsis screening method.

**Figure 1.**
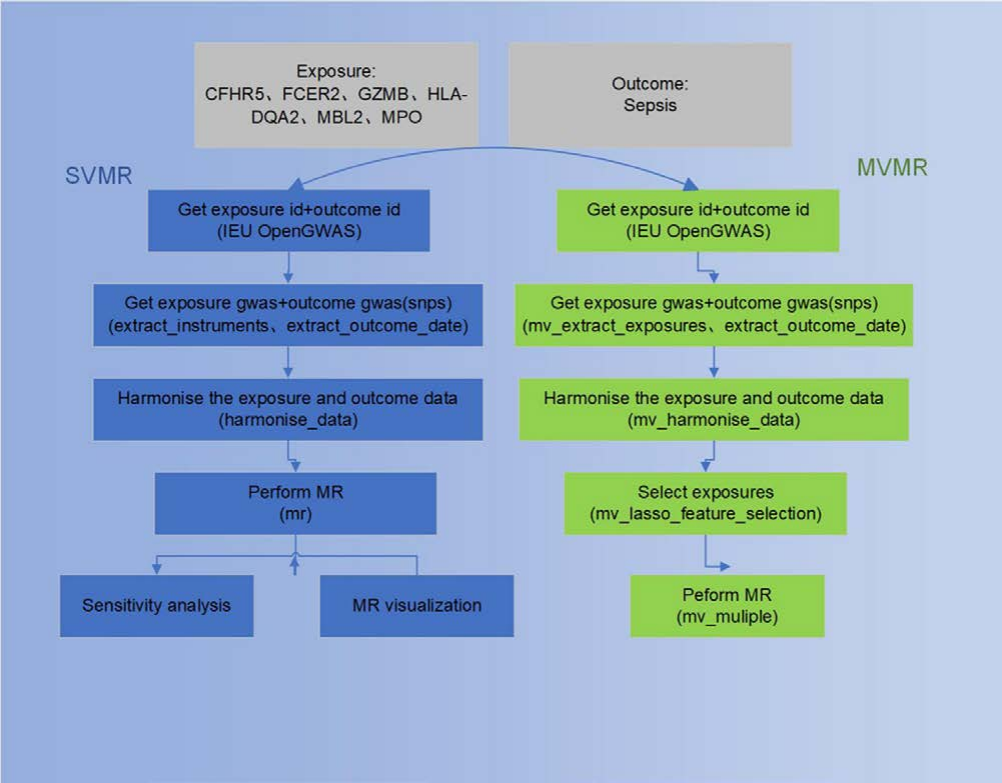
Study design for our MR. CFHR5 = complement factor H-related protein-5, FCER2 = Fc epsilon receptor II, GWAS = genome-wide association study, GZMB = granzyme B, HLA-DQA2 = major histocompatibility complex, class II, DQ alpha, id = Identification, IEU = Integrative Epidemiology Unit, MBL2 = mannose-binding lectin 2, MPO = myeloperoxidase, MR = Mendelian randomization, MVMR = multivariate Mendelian randomization, SVMR = single-variable Mendelian randomization.

Based on single-nucleotide polymorphism (SNP) data on sepsis and immune protein exposure factors from the genome-wide association study (GWAS) public database, this study conducted analyses using univariate MR (UVMR) and multivariate MR (MVMR) to investigate the causal influence between immune effector–related proteins and sepsis. Additionally, we conducted sensitivity analyses to assess the impact of assumptions on findings and to ensure the robustness of results. Our study provides guidance for elucidating immune effector proteins in sepsis pathogenesis. Figure 1 provides a detailed overview of the study.

## 2. Materials and methods

### 2.1. Data source

GWAS data for sepsis (ieu-b-5086), complement factor H-related protein-5 (CFHR5) (prot-a-521), Fc epsilon receptor II (FCER2) (prot-a-1072), GZMB (prot-a-1299), major histocompatibility complex, class II, DQ alpha (HLA-DQA2) (prot-a-1347), MBL2 (prot-a-1863), and myeloperoxidase (MPO) (prot-a-1930) were being collected via the Integrative Epidemiology Unit Open About Sepsis GWAS database (https://gwas.mrcieu.ac.uk/). From 486,484 samples, 12,243,487 SNPs were included in the sepsis dataset. Additionally, each of the 6 immune effector–associated proteins included 10,534,735 SNPs from 3301 samples. All data were derived from the European population (Table 1).

**Table 1:** The GWAS data source details in our study.

Exposure	ID	SNP	Sample	Population
prot-a-1072	FCER2	10,534,735	3301	European
prot-a-1299	GZMB	10,534,735	3301	European
prot-a-1347	HLA-DQA2	10,534,735	3301	European
prot-a-1863	MBL2	10,534,735	3301	European
prot-a-1930	MPO	10,534,735	3301	European
prot-a-521	CFHR5	10,534,735	3301	European
ieu-b-5086	Sepsis	12,243,487	486,484	European

CFHR5 = complement factor H-related protein-5, FCER2 = Fc epsilon receptor II, GWAS = genome-wide association study, GZMB = granzyme B, HLA-DQA2 = major histocompatibility complex, class II, DQ alpha, ID = Identification, MBL2 = mannose-binding lectin 2, MPO = myeloperoxidase, SNP = single-nucleotide polymorphism.

### 2.2. Data processing

To extract IVs with noteworthy connections to exposure factors (CFHR5, FCER2, GZMB, HLA-DQA2, MBL2, or MPO), the extract_instruments function of R package TwoSampleMR (v 0.5.6) and *P* < 5 × 10^−8[12]^ were applied. When the conditions clump = TRUE, r^2^ = 0.001, and kb = 10,000 were fulfilled, IVs for linkage disequilibrium were eliminated. Finally, after screening the IVs, the repeated and sepsis-related SNPs were removed from the UVMR inputs.

Both UVMR and MVMR utilized the same data preprocessing techniques. The covariate screening variables were then removed with the mv_lasso_feature_selection function, yielding IVs linked to any of the 6 proteins but unrelated to sepsis as MVMR inputs for analysis.

### 2.3. UVMR analyses

Using weighted median,^[13]^ MR-Egger,^[14]^ simple mode,^[15]^ inverse-variance weighted (IVW),^[15]^ and weighted mode^[16]^ approaches, UVMR investigated the relationship between CFHR5, FCER2, GZMB, HLA-DQA2, MBL2, or MPO and sepsis. Additionally, with a focus on the IVW method’s findings, the study examined the causal relationship between the aforementioned variables and sepsis. The computed odds ratio (OR) value of 1 was then applied as a cutoff, where a value of >1 indicated a risk factor and a value <1 indicated a protective factor.

### 2.4. Sensitivity tests and MVMR analyses

Sensitivity experiments involving the heterogeneity test (Cochran Q),^[17]^ horizontal pleiotropy,^[18]^ and leave one out (LOO)^[19]^ were conducted in order to evaluate the robustness of UVMR results. Finally, 6 immune effector–related proteins’ genetic variables were investigated in MVMR to further examine their effects on sepsis.

## 3. Results

### 3.1. The MBL2 protein was determined to be a risk factor for sepsis when compared to 5 other immune-related proteins

Following the IV screening, we identified SNPs linked to the proteins and SNPs among these SNPs that were not linked to sepsis. In addition, for UVMR analysis, FCER2, GZMB, and CFHR5 all gained 3 separate SNPs, whereas HLA-DQA2, MBL2, and MPO all picked up 4 independent SNPs. All 6 of the proteins with sepsis had *P* values <0.05 in the IVW results (FCER2: *P* = .002; GZMB: *P* < .001; CFHR5: *P* = .003; HLA-DQA2: *P* = .025; MBL2: *P* = .005; MPO: *P* = .006), suggesting a causal relationship between them and sepsis. Concretely, the evaluation of OR values revealed further information, indicating that the MBL2 (OR = 1.046) protein was a risk factor for sepsis, whereas the other proteins were safety factors (FCER2: OR = 0.922; GZMB: OR = 0.908; CFHR5: OR = 0.858; HLA-DQA2: OR = 0.896; MPO: OR = 0.875).

**Figure 2.**
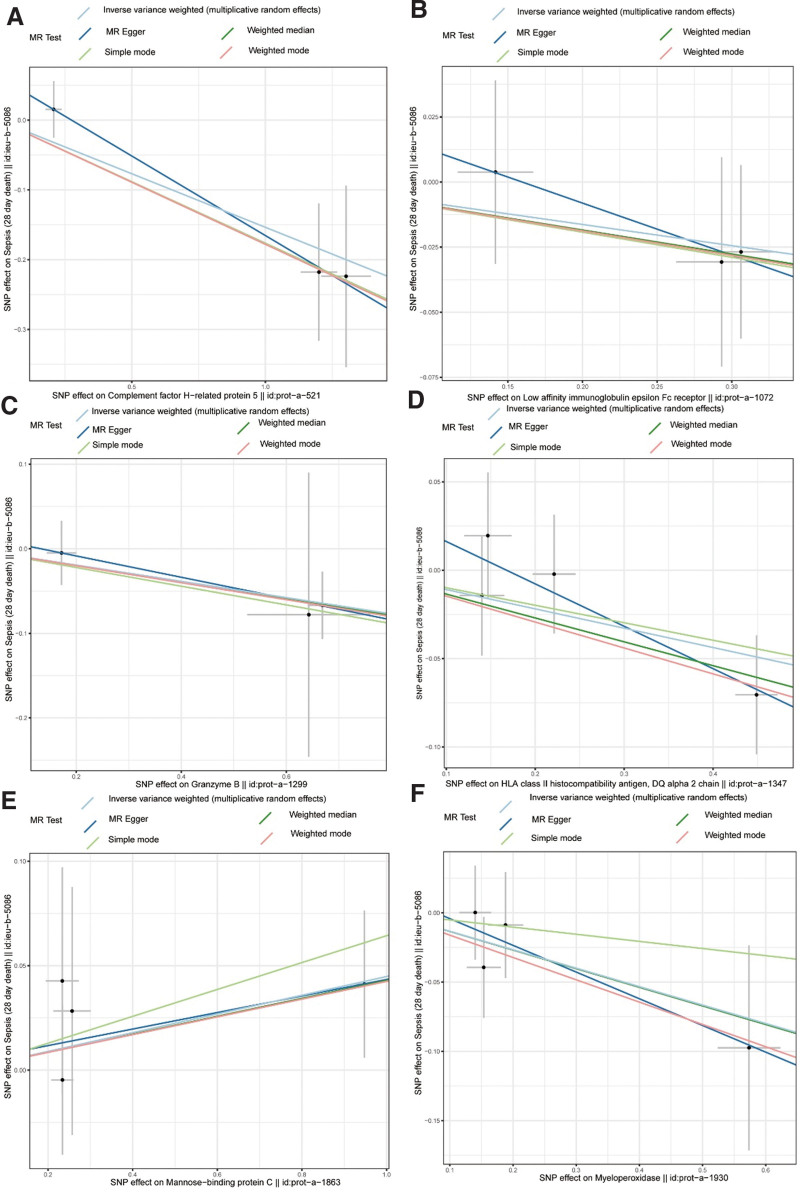
Scatter plot of the correlation between 6 immune effector proteins and sepsis. (A) CFHR5, (B) FCER2, (C) GZMB, (D) HLA-DQA2, (E) MBL2, (F) MPO. CFHR5 = complement factor H-related protein-5, DQ = linkage disequilibrium, FCER2 = Fc epsilon receptor II, GZMB = granzyme B, HLA-DQA2 = major histocompatibility complex, class II, DQ alpha; MBL2 = mannose-binding lectin 2, MPO = myeloperoxidase, MR = Mendelian randomization, SNP = single-nucleotide polymorphism.

**Figure 3.**
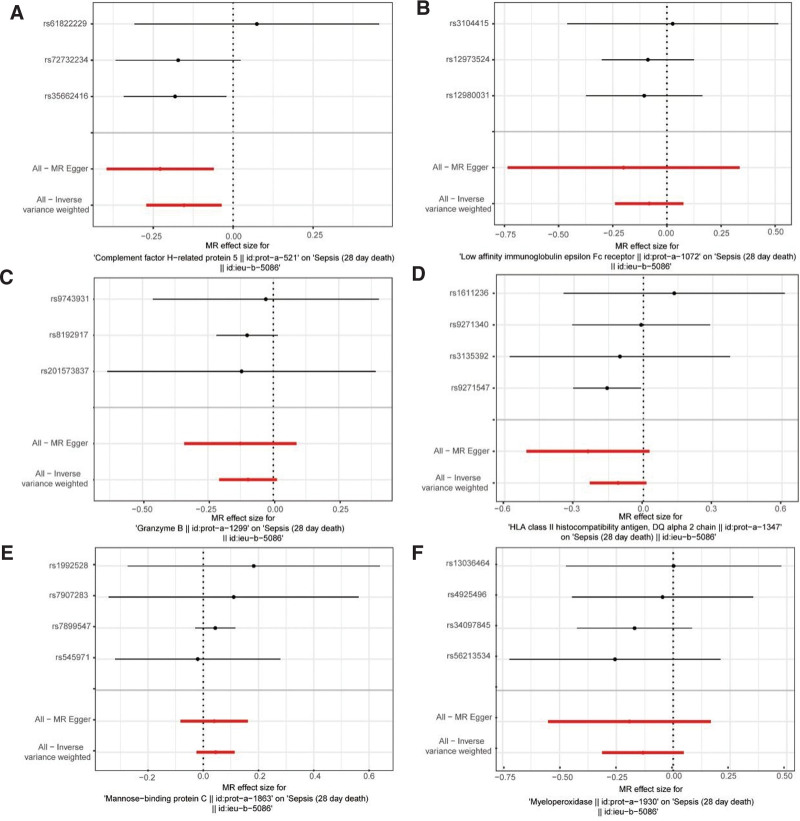
Forest plot of the UVMR effect of each of the 6 immune effector proteins with sepsis. (A) CFHR5, (B) FCER2, (C) GZMB, (D) HLA-DQA2, (E) MBL2, (F) MPO. CFHR5 = complement factor H-related protein-5, FCER2 = Fc epsilon receptor II, GZMB = granzyme B, HLA-DQA2 = major histocompatibility complex, class II, DQ Alpha, MBL2 = mannose-binding lectin 2, MPO = myeloperoxidase, MR = Mendelian randomization, UVMR = univariate Mendelian randomization.

**Figure 4.**
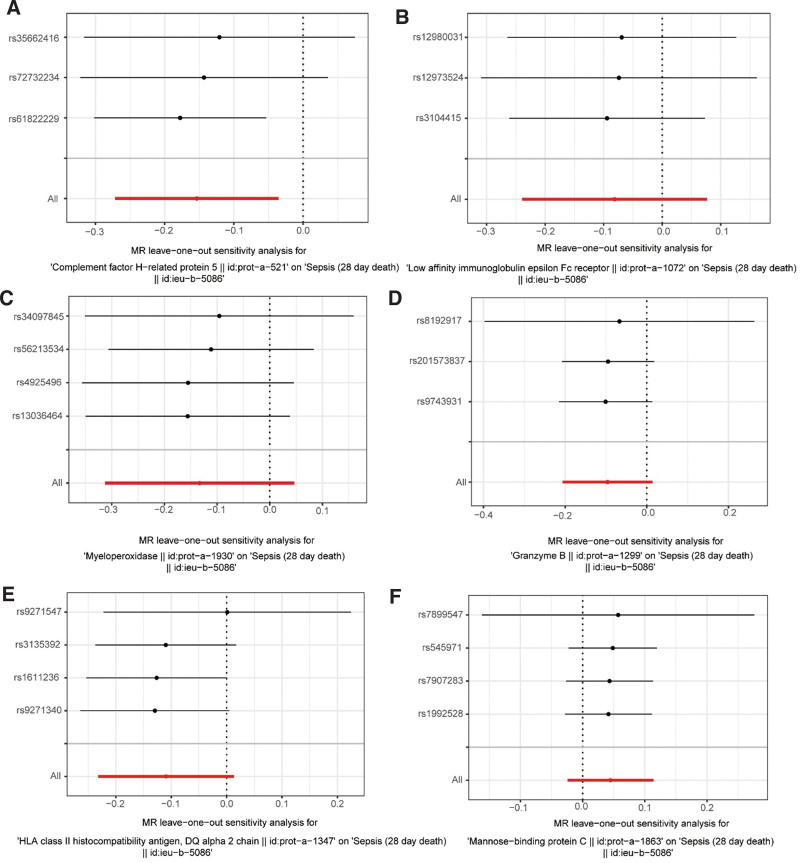
The LOO test detects the effect of independent SNPs on MR results. (A) CFHR5, (B) FCER2, (C) MPO, (D) GZMB, (E) HLA-DQA2, (F) MBL2. CFHR5 = complement factor H-related protein-5, DQ = linkage disequilibrium, FCER2 = Fc epsilon receptor II, GZMB = granzyme B, HLA-DQA2 = major histocompatibility complex, class II, DQ alpha, LOO = leave one out, MBL2 = mannose-binding lectin 2, MPO = myeloperoxidase, MR = Mendelian randomization, SNP = single-nucleotide polymorphism.

Examining the impacts of SNPs on exposure factors and sepsis in a scatter plot, respectively, we observed that the MBL2 protein had a positive slope, implying that it was a risk factor for sepsis, whereas the remaining 5 proteins displayed the opposite pattern (Fig. 2A–F). The findings of forest plot demonstrated that the MR effect size of MBL2 on sepsis was >0 and that of the other proteins was <0, further illuminating the immune effector–related proteins’ causative roles in sepsis (Figs. 3A–4F). Mendel’s second law of randomized grouping was followed by all UVMR data, as demonstrated by the final funnel plots (Figures S1A–S4F, Supplemental Digital Content, http://links.lww.com/MD/N467).

### 3.2. The UVMR results’ durability was proven by sensitivity testing

The heterogeneity test, horizontal pleiotropy, and LOO were utilized to verify the robustness of UVMR findings, in that order. It proved that none of the IVs were heterogeneous across their respective datasets and sepsis dataset, with *P* values for all 6 proteins in the heterogeneity test being >0.05 (FCER2: *P* = .895; GZMB: *P* = .948; CFHR5: *P* = .470; HLA-DQA2: *P* = .610; MBL2: *P* = .894; MPO: *P* = .840). Then, the horizontal pleiotropy test similarly had a *P* value >0.05 (FCER2: *P* = .728; GZMB: *P* = .805; CFHR5: *P* = .436; HLA-DQA2: *P* = .392; MBL2: *P* = .928; MPO: *P* = .742), suggesting that all IVs influenced sepsis via each of the 6 exposure factors and not via other channels (Table 2). Ultimately, the LOO indicated that UVMR results were not significantly influenced by any particular SNP (Fig. 4A–F). To sum up, there was strong stability in the UVMR data.

**Table 2:** Heterogeneity test between each of the 6 immune effector proteins and the sepsis dataset.

Outcome	Exposure	ID	Heterogeneity	Horizontal pleiotropy
ieu-b-5086	prot-a-1072	FCER2	0.89494	0.728433
ieu-b-5086	prot-a-1299	GZMB	0.947814	0.804777
ieu-b-5086	prot-a-1347	HLA-DQA2	0.610449	0.392012
ieu-b-5086	prot-a-1863	MBL2	0.893521	0.927984
ieu-b-5086	prot-a-1930	MPO	0.839512	0.742007
ieu-b-5086	prot-a-521	CFHR5	0.469857	0.435956

CFHR5 = complement factor H-related protein-5, FCER2 = Fc epsilon receptor II, GZMB = granzyme B, HLA-DQA2 = major histocompatibility complex, class II, DQ alpha, ID = Identification, MBL2 = mannose-binding lectin 2, MPO = myeloperoxidase.

### 3.3. The 6 immune effector–related proteins’ causative roles in sepsis were further validated by the MVMR data

**Figure 5.**
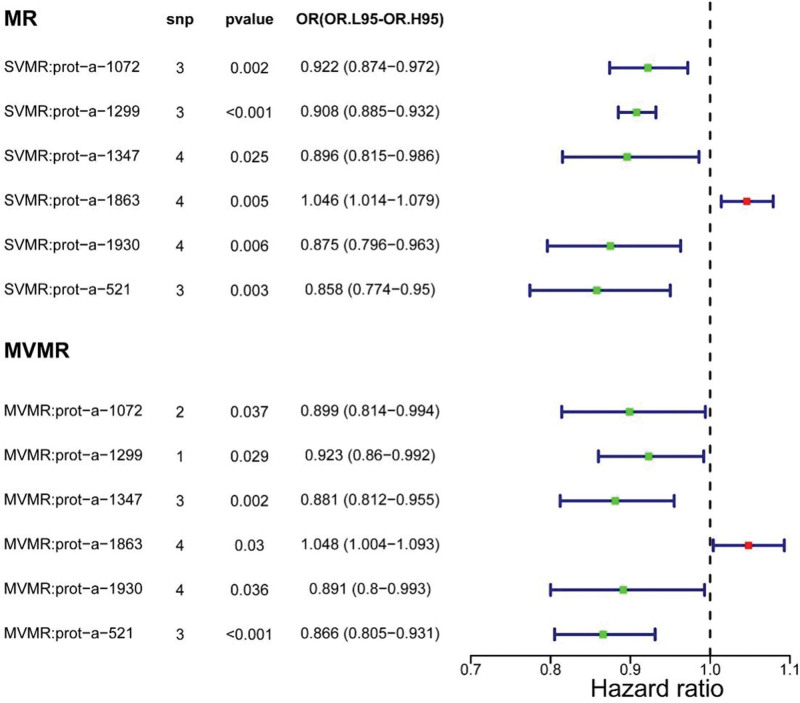
Forest plot for the causal effect of immune effector proteins on the risk of sepsis derived from IVW. IVW = inverse-variance weighting, MR = Mendelian randomization, MVMR = multivariate Mendelian randomization, OR = odds ratio, SVMR = single-variable Mendelian randomization.

In order to explore the causality of 6 exposure factors on sepsis at multivariate level, we conducted MVMR analysis. First, screening produced a total of 17 SNPs that were linked to any of the 6 proteins but not sepsis. Afterward, we discovered that both the *P* values of exposure factors were <0, and the OR value of MBL2 was >1, while the OR values of the remaining proteins were <1, which was consistent with the UVMR results (Fig. 5). In conclusion, MBL2, FCER2, GZMB, HLA-DQA2, MPO, and CFHR5 all played key roles in the development of sepsis, with elevated MBL2 protein expression increasing the likelihood of sepsis and the remaining 5 proteins having the reverse effects.

## 4. Discussion

Sepsis has a high mortality rate and poses a major threat to human life and health.^[20]^ However, studies on the complex mechanisms between sepsis and immune effects are still insufficient.^[21]^ During sepsis pathogenesis, this MR study investigated the causal relationship between immune effector proteins and sepsis. The study of immune effector proteins may lead to new therapeutic strategies for treating and preventing sepsis. In the results of this article, we found that MBL2 is a risk factor for sepsis, whereas FCER2, GZMB, HLA-DQA2, MPO, and CFHR5 are protective factors for sepsis.

MBL2, produced by hepatocytes and secreted into the bloodstream, is a protein involved in the natural immune response. It recognizes specific glycan structures on the surface of bacteria, viruses, and fungi, either directly contributing to their clearance or indirectly participating in pathogen clearance by activating the complement system.^[22]^ Early stages of the SARS-CoV-2 infection significantly elevate MBL2 levels, leading to hyperactivation of the complement system and triggering more severe lung disease.^[23]^ Furthermore, studies have observed that the *MBL2* gene frequently carries D, B, and C mutations. These mutations reduce MBL2 activity, weaken complement activation, and result in incomplete pathogen clearance, ultimately leading to more severe disease.^[24]^ Therefore, we speculate that the overactivation of the complement system, along with possible variants in the *MBL2* gene, could be the reason why MBL2 is a risk factor for sepsis.

It was noted that FCER2, an IgE receptor protein expressed primarily on the surface of B cells, regulates the inflammatory process by influencing inflammatory cell migration and activation. In addition, it regulates the activity of B cells and IgE, which are involved in preventing immune attacks against tissues.^[25]^ Cytotoxic T cells and natural killer cells produce GZMB, a serine protease. It controls inflammation by promoting apoptosis of inflammatory cells, attenuates tissue damage, and protects tissues from excessive inflammation, as noted in the study by Hirota et al.^[26]^ In a mouse model of acute viral infection, we discovered that GZMB maintains immune homeostasis by suppressing immune system activity and preventing excessive inflammatory responses and autoimmune diseases via regulatory T cells.^[27]^ Within the major histocompatibility complex class II region, the *HLA-DQA2* gene, part of the *HLA-DQ* gene family, codes for an α-chain. It helps to maintain its own components’ immune tolerance and prevent attacks on its own tissues.^[28]^

Neutrophils and monocytes produce the enzyme MPO.^[29]^ Hepatocytes also produce CFHR5, which is a component of the complement system. The complement system forms membrane attack complexes that disrupt pathogen cell membranes and generates a series of small molecule cleavage products such as C3a and C5a to protect its own tissues from excessive complement activation.^[30]^ CFHR5 has a role in controlling complement activation.^[31]^ In summary, FCER2, GZMB, HLA-DQA2, MPO, and CFHR5 are protective factors in the pathogenesis of sepsis because they regulate various aspects of the immune response, thereby preventing overactivation of inflammation and protecting self-tissues from damage.

In this study, we used MR to establish causal inference and minimize the effect of confounding bias.^[8]^ This study explores the relationship between 6 immune effector proteins and sepsis, providing a valuable resource for understanding new pathological mechanisms. Furthermore, it is the first study to use MR analysis to investigate immune effector proteins and sepsis risk. However, the study still has many limitations. First, the study represents a population of European origin; to determine whether the findings are equally applicable to all population groups, further studies are necessary. In addition, we were unable to perform subgroup analyses, such as how to distinguish between early-onset and late-onset sepsis. Finally, we also lack some mechanistic studies to support our results. Therefore, further studies are necessary to clarify the connection between immune effector proteins and sepsis.

## 5. Conclusions

In conclusion, our findings demonstrate that immune effector proteins have a genetic basis for their effect on sepsis. This study also provides useful information for assessing sepsis risk, researching sepsis pathophysiology and immunological effects, and developing preventive and treatment techniques.

## Author contributions

**Conceptualization:** Xiaoqiong Wang.

**Data curation:** Xiaona Yin, Xi Wang.

**Formal analysis:** Yao Zhang, Lijuan Zhou.

**Methodology:** Ting Zhang.

**Supervision:** Fangbing Du.

**Validation:** Yinling Jiang.

**Visualization:** Chuchu Xu.

**Writing – original draft:** Yongsheng Wang, Xiaoqiong Wang.

**Writing – review & editing:** Yongsheng Wang, Xiaoqiong Wang.

## Supplementary Material


