# Supplementary figures and images for: Causal association of immune effector proteins with sepsis: A Mendelian randomization study
Source: Medicine (Baltimore). 2024 Sep 6;103(36):e39494. doi: 10.1097/MD.0000000000039494 (PMC11384063; doi:10.1097/MD.0000000000039494)

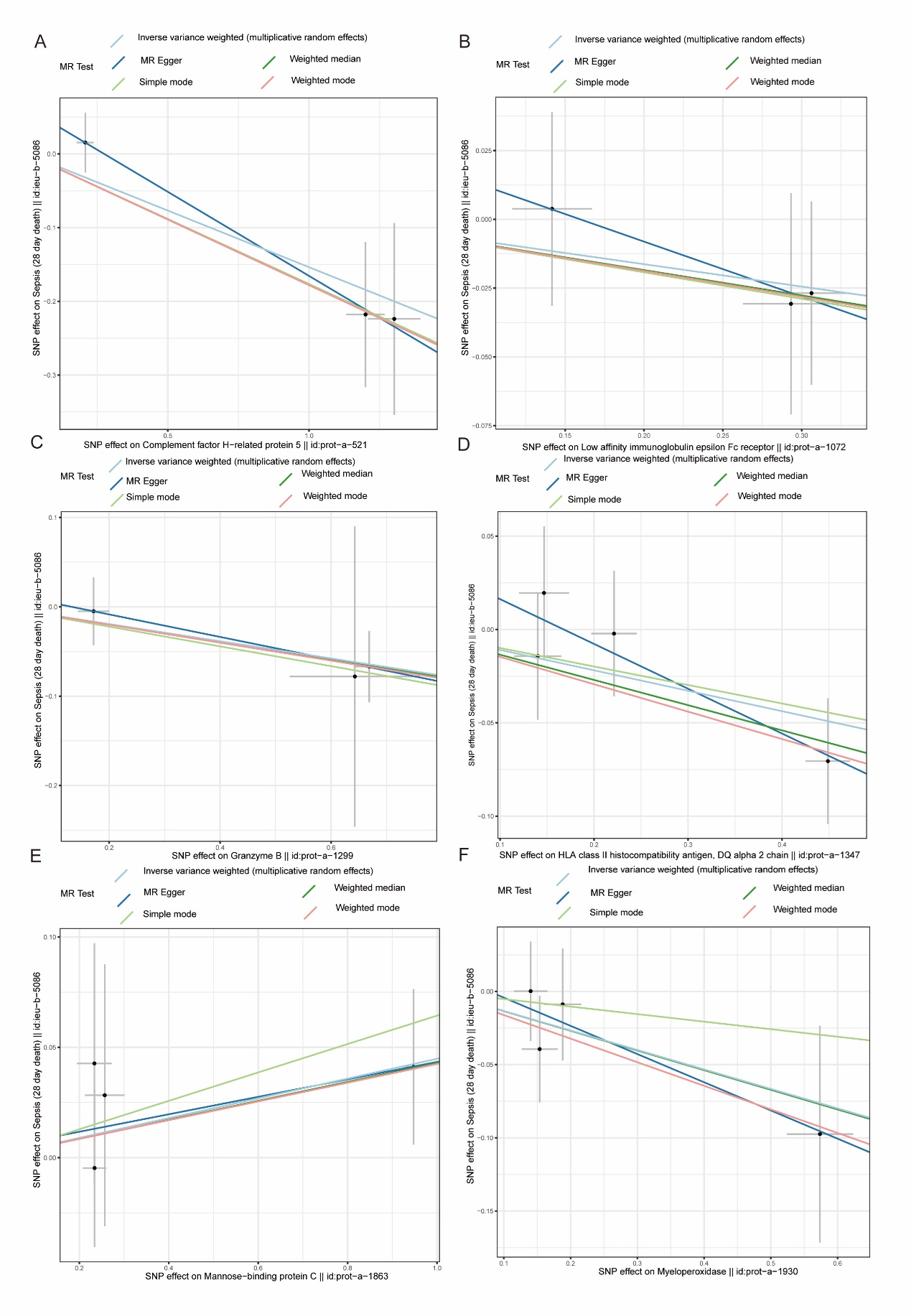

Supplement: Supplementary file 1 [file medi-103-e39494-s001.tif]
